# Association between Parkinson’s disease and risk of prostate cancer in different populations: An updated meta-analysis

**DOI:** 10.1038/s41598-017-13834-x

**Published:** 2017-10-18

**Authors:** Chunli Chen, Haiping Zheng, Zhiping Hu

**Affiliations:** Department of Neurology, Second Xiangya Hospital, Hunan, P. R. China

## Abstract

Recently, growing evidence has revealed a significant association between Parkinson’s disease (PD) and cancer. However, controversy still exists concerning the association between PD and prostate cancer. A comprehensive article search for relevant published studies was performed using the online databases PubMed, Web of Science and Embase up to January 1, 2017. The pooled risk ratios (RRs) and their 95% confidence intervals (CIs) were calculated using the method of inverse variance with a random-effects model. Fifteen studies comprising 346,153 PD patients were included in this study. The results of the present study showed that PD was significantly associated with a decreased risk of prostate cancer in the Western population (RR: 0.83, 95% CI: 0.72–0.95, P < 0.01), while an increased risk of prostate cancer was shown in the Asian population (RR: 1.80, 95% CI: 1.52–2.13, P < 0.001). In the subgroup analysis, the reduced risk of prostate cancer in PD patients from Western populations was consistent regardless of study design or study quality. In conclusion, PD was significantly associated with a reduced risk of prostate cancer in the Western population. The relationship between those conditions in the Asian population needs to be confirmed by future studies.

## Introduction

Parkinson’s disease (PD) is the second most common neurodegenerative disorder in the world, with a prevalence of 1% in the population over 60 years old^1^. Resting tremor, rigidity, hypokinesia, and postural instability are considered the four cardinal motor symptoms of PD, resulting from the loss of dopaminergic neurons in the substantia nigra pars compacta^2^. However, the pathogenesis of PD is not yet fully understood.

In recent years, accumulating epidemiological and clinical studies have reported the relationship between PD and cancer, lighting the way to explore the potential common pathogenic pathway involved in both diseases^3^. It has been noted that cancer rates are lower in patients with Parkinson’s disease than in the general population, and PD has different relationships with the risk of different cancers^4^. For instance, Wang *et al*.^5^ reported a lack of association between PD and prostate cancer in a previous meta-analysis. However, its conclusions were not very convincing owing to the relatively small sample size, limited number of studies and duplicate population of some included studies. Controversy still exists regarding the relationship between PD and prostate cancer. Lately, some articles have reported that patients with PD have a decreased risk of prostate cancer^6,7^, while some have found no significant negative association between them^8,9^, and some even hold the opposite opinion and indicate that an increased risk of prostate cancer could be observed among people with PD^10^. On the other hand, there is some potential evidence to link PD to prostate cancer. The most commonly used medications for Parkinson’s disease are levodopa, dopamine agonists and anticholinergics^11^, which all affect neurotransmitter activity, and it is possible that these treatments may affect tumor occurrence.

Indeed, differences in characteristics such as ethnicity, study design, PD diagnosis time and PD treatment have led to discrepancies in estimates of the association between Parkinson’s disease and risk of prostate cancer. Therefore, we conducted this meta-analysis to provide a quantitative assessment of current epidemiological evidence on the association between PD and risk of prostate cancer in different subgroups and explore the potential factors which can affect the association.

## Results

### Eligible studies

**Figure 1:**
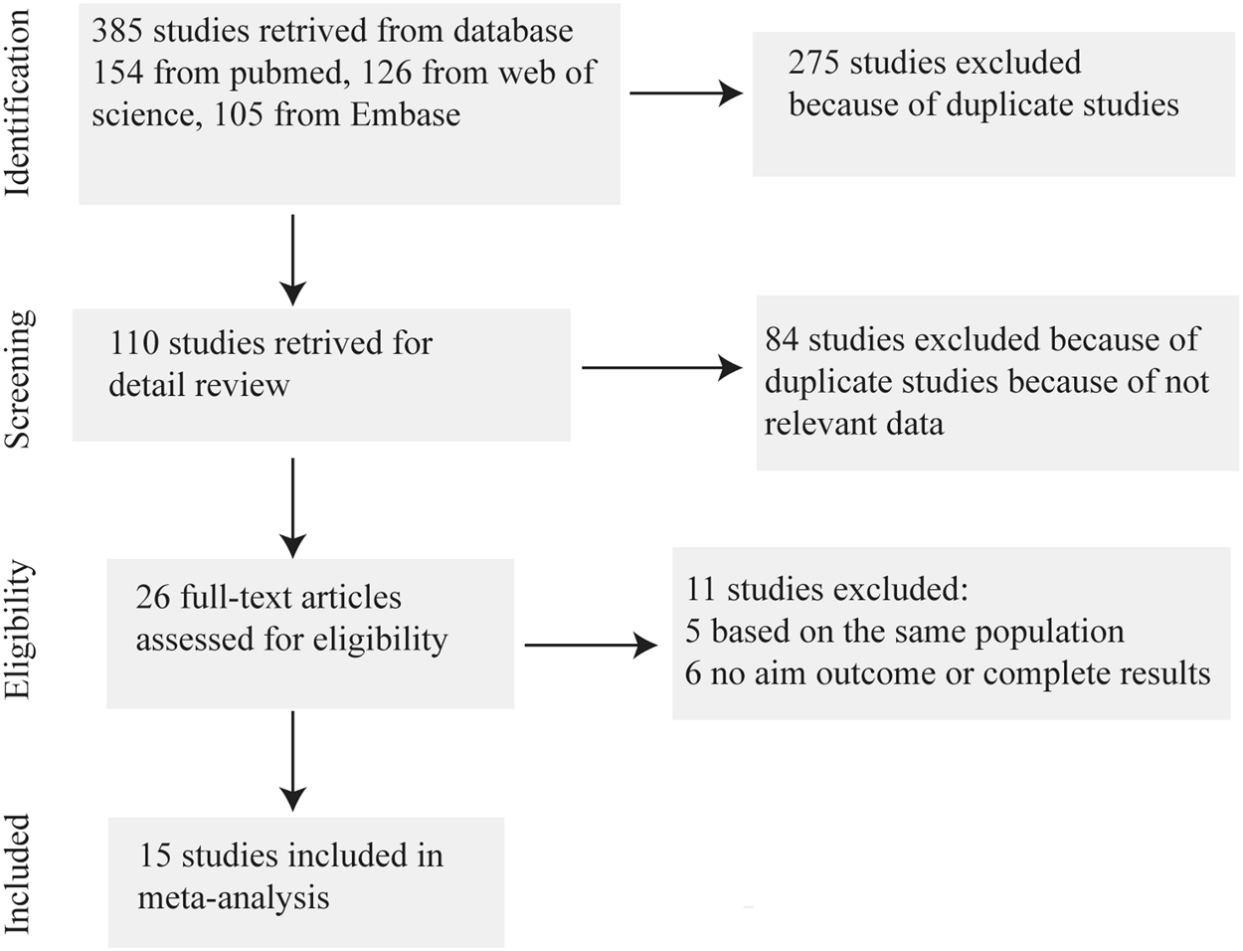
Flow diagram of study selection procedure.

A total of 385 potentially relevant studies were identified in the database search. After exclusion of 275 duplicated studies, 110 studies remained. After title and abstract review, 84 studies were excluded. After full-text review of the remaining 26 studies, 5 duplicated studies that were performed in the same population and 6 studies without relevant outcomes of interest or without complete results were excluded. Ultimately, 15 studies were included in our meta-analysis^6–10,12–21^ (Fig. 1).

These studies were published between 2002 and 2016 and included 346,153 patients with PD. Among these 15 studies, 1 study were performed in an Asian population, while 14 studies were conducted in Western populations (6 in the USA, 3 in the UK, 1 in Canada, 2 in Denmark, 1 in Israel and 1 in Sweden). The baseline characteristics of each study are shown in Table 1.

**Table 1:** Baseline characteristics of included studies in different populations.

Author	Year	Country/Area	Study design	Number of case, source	Number of control, source	Tumor identification	Adjustment	OI	SQ	Ethnicity
Elbaz	2002	USA	Case-control	196, Rochester Epidemiology Project	196, general population	Before	Age, sex	OR	7	Western population
Guttman	2004	Canada	Cohort	15306, OHIP, ODB RPDB	30612, general population	After	Age, sex	RR	6	Western population
Powers	2006	USA	Case-control	352, clinics of Group Health Cooperative	484, enrollees of Group Health Cooperative	Before	Age, sex, year of enrollment, geographical location.	OR	6	Western population
Driver	2007	USA	Cohort	572, Physician’s Health Study	487, Physician’s Health Study	After	Age	RR	6	Western population
Fois	2010	UK	Cohort	4355, UK National Health Service hospitals	NR, UK National Health Service hospitals	Before/after	Age, sex, calendar year of first recorded admission	RR	7	Western population
Lo	2010	USA	Cohort	692, Kaiser Permanente Northern California Medical Care Plan	761, Kaiser Permanente Northern California Medical Care Plan	Before/after	Age, sex, cigarette smoking, alcohol consumption, body mass index	RR	7	Western population
Becker	2010	UK	Cohort	2993, UK-based General Practice Research Database	3003, UK-based General Practice Research Database	After	Age, sex, general practice, diagnosis date, years of history	IRR	6	Western population
Rugbjerg	2012	Denmark	Cohort	20343 Danish Hospital Register	NR, general population	After	Age, sex, calendar period	SIR	7	Western population
Wirdefeldt	2013	Sweden	Cohort	11786, Swedish Patient Register	58930, Swedish Patient Register	Before/after	Age. sex	HR	7	Western population
Ong	2014	UK	Cohort	219194, English national Hospital Episode Statistics	9015614, English national Hospital Episode Statistics	After	Age, sex, calendar year, region of residence, quintile of patients	RR	8	Western population
Lin	2015	Taiwan	Cohort	62023, National Health Insurance	124046, National Health Insurance	After	Age, sex	HR	7	Asian population
Peretz	2016	Israel	Cohort	7125, Maccabi Health Services	NR, Maccabi Health Services	After	Age, sex, chronological year	SIR	7	Western population
Tacik	2016	USA	Case-control	971, Mayo Clinic	478, Mayo Clinic	Before	Age, sex	OR	5	Western population
Freedman	2016	USA	Case-control	NR, SEER-Medicare	NR, SEER-Medicare	After	Age, sex, selection year	OR	5	Western population
Jespersen	2016	Denmark	Case-control	245, Danish Civil Registration System	1656, Danish Civil Registration System	After	Age, sex. index date	OR	7	Western population

HR: hazard ratio; IRR: incidence rate ratio; NR: not reported; ODB: Ontario Drug Benefit; OHIP: Ontario Health Insurance Plan; OR: odds ratio; OI: outcome of interest; RPDB: Registered Persons Database; RR: relative risk; SEER: Surveillance, Epidemiology, and End Results; SIR: standardized incidence ratio; SQ: score of study quality. Study quality was judged based on the Newcastle-Ottawa Scale.

### PD and risk of prostate cancer

**Figure 2:**
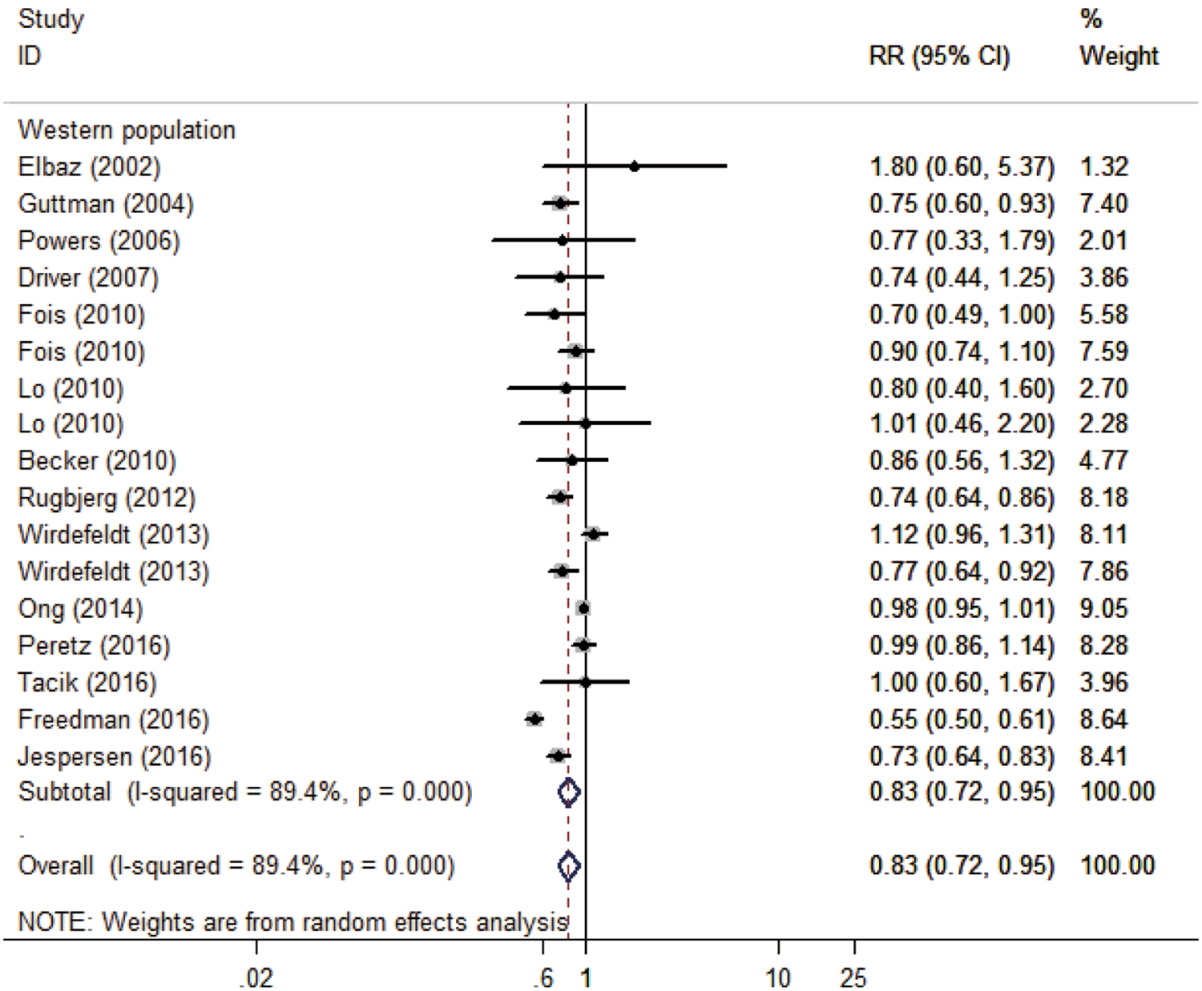
Forest plot of risk ratio for the association between Parkinson’s disease and risk of prostate cancer in different populations. OR, odds ratio; CI, confidence interval.

The result of 10 cohort studies and 5 case-control studies indicated that the pooled RR of prostate cancer in PD patients versus control patients was 0.88 (95% CI: 0.76–1.02, P = 0.082, I^2^ = 92%, Fig. 2), thus showing that PD patients had no significant risk of prostate cancer compared with the general population.

In addition, we excluded one study that was conducted in an Asian population^10^ and performed further analyses in Western populations. The pooled result showed that PD was significantly associated with a decreased risk of prostate cancer in Western populations (RR: 0.83, 95% CI: 0.72–0.95, P < 0.01, I^2^ = 85%, Table 2). Furthermore, subgroup analyses revealed that the significance of the inverse association between PD and risk of prostate cancer in Western populations was not affected by study design and study quality (Table 2).

**Table 2:** Subgroup analyses for association between Parkinson’s disease and the risk of prostate cancer in Western population.

Categories	N	Pooled RR	95% CI	P value	Heterogeneity	
					I2 (%)	P′
Overall effect	14	0.83	(0.72, 0.95)	0.007	89.4%	<0.001
**Study design**						
Cohort	9	0.88	(0.80, 0.97)	0.008	66.7%	<0.001
Case-control	5	0.73	(0.56, 0.94)	0.016	78.4%	<0.001
**Study quality**						
>6	8	0.88	(0.78, 0.98)	0.019	78.0%	<0.001
<6	6	0.72	(0.58, 0.91)	0.005	64.9%	0.014
**The diagnosis time of Prostate cancer**						
Before PD	6	1.03	(0.92, 1.16)	0.624	0.0%	0.506
After PD	11	0.77	(0.65, 0.92)	0.003	93.0%	<0.001

CI: confidence interval; N: number of studies; RR: risk ratio; PD: Parkinson’s disease. P’: p value of Q test for heterogeneity.

Furthermore, according to the diagnosis time of prostate cancer, we divided the studies into “before PD” and “after PD” groups to perform subgroup analysis in the Western population. The result indicated that a significant inverse relationship between PD and the risk of prostate cancer could be found in the “after PD group” (RR: 0.77, 95% CI: 0.65–0.92, P < 0.01), but it could not be found in the “before PD” group (RR: 1.03, 95% CI: 0.92–1.16, P = 0.62, Table 2).

### Sensitivity analysis

In our meta-analysis, sensitivity analysis was used to assess the stability of the results. The significant inverse association between PD and risk of prostate cancer in the Western population did not change in the sensitivity analysis, which was conducted by removing each study in turn (Supplementary Figure S1).

### Cumulative meta-analysis

We used cumulative meta-analysis to evaluate the association between PD and prostate cancer risk in relation to publication year. Our result indicated that from 2007 to present, the significant inverse relationship between PD and risk of prostate cancer in the Western population remained consistent (Supplementary Figure S2).

### Publication bias analysis

In our meta-analysis, we used Begg’s and Egger’s tests to evaluate the effect of publication bias. There was no obvious evidence of publication bias as revealed by Begg’s funnel plots (Begg, P = 0.232, Supplementary Figure S3A). However, publication bias was detected by Egger’s regression test (Egger, P = 0.007, Supplementary Figure S3B).

## Discussion

PD is characterized by the significant loss of dopaminergic neurons in the substantia nigra pars compacta and the presence of intraneuronal proteinaceous cytoplasmic inclusions termed Lewy bodies^22^, but the etiology of the disease is still poorly understood. Although PD and cancer seem to drive the cells to different outcomes, that is, either degeneration or overproliferation, the association between PD and cancer has been supported by plenty of epidemiologic studies, which have shown that the incidence rates of most cancers are lower in PD patients than in controls^4,23^.

In this study, the pooled result in all populations indicated that PD patients had no significant risk of developing prostate cancer. However, PD was significantly associated with a decreased risk of prostate cancer in the Western population, while an increased risk of prostate cancer was indicated in the Asian population. The significance of the inverse association between PD and risk of prostate cancer in the Western population was unaffected by the factors of study design and study quality. The results of sensitivity analysis and cumulative meta-analysis also confirmed the robustness of the relationship between them. On the other hand, the results from the Asian population showed almost the opposite findings compared with the studies performed in Western populations. This discrepancy may be explained by different genetic backgrounds^24,25^ and different environmental exposures^26,27^, which play important roles in a diverse array of disease pathogenetic processes. Furthermore, only one study^10^ was performed in an Asian population, and this seemed far more likely to represent the combined effect of publication and reporting bias than an actual underlying association in the Asian population. More studies are warranted to investigate the association between Parkinson’s disease and prostate cancer in the Asian population in the future.

Possible mechanisms of the significant negative association between PD and prostate cancer in the Western population are as follows. First, the different essential characteristics of these two diseases may be one explanation. PD is a neurodegenerative disorder characterized by dopaminergic neuronal death, whereas prostate cancer is a disease characterized by unlimited cell proliferation and lack of apoptosis. Cells in PD patients may be more likely to undergo apoptosis to fight against the progression of cancer^9^. The second explanation for this discrepancy could be consequences of the pharmacological treatment of Parkinson’s disease, which affects neurotransmitter activity. The main goal of medical treatment of Parkinson’s disease is to increase the amount of dopamine in the central nervous system; this dopamine can ultimately be converted to adrenalin, which stimulates the sympathetic nervous system^28^. Additionally, anticholinergic drugs, used in the treatment of Parkinson’s disease, can stimulate the parasympathetic nervous system^29^. Further research showed that stimulation of newly formed sympathetic nerves and parasympathetic nerve fibers in the autonomic nervous system can promote early stages of tumor genesis and then promote cancer dissemination^30^. Third, previous studies reported that smoking conferred a decreased risk of PD^13^ and a modestly increased risk of prostate cancer^31^. Thus, smoking may also partly explain the significant inverse association between PD and the risk of prostate cancer.

This study has several strengths. We involved more eligible evidences and carefully assessed the quality of evidence, which made the results much more reliable, and we also excluded duplicate studies that were based on the same population. Moreover, the results of this study in the Western population are robust, as shown by further subgroup analysis, sensitivity analysis and cumulative meta-analysis. Several limitations should be considered in the interpretation of our results. First, some of the included studies in our analysis were retrospective cohort studies or case-control studies. Second, subgroup analyses were not performed according to factors such as gender, age, smoking and alcohol consumption because insufficient data were extracted from the primary articles. Third, publication bias and other forms of bias may have existed in our results due to limitations in the inclusion criteria.

## Methods

### Literature search strategy

A comprehensive article search for relevant published studies was performed using the following online databases: PubMed, Web of Science and Embase.

The main search terms “(Parkinson* OR parkinson’s disease OR Parkinsons disease OR Parkinson disease) AND (prostate cancer or prostate cancers or prostate neoplasm or prostate neoplasms or prostate carcinoma or prostate carcinomas)” were used to search for relevant studies published up to January 1, 2017. Moreover, the references of relevant reviews and included articles were also carefully reviewed to identify additional studies that might be suitable for inclusion.

### Study selection and data extraction

The eligible studies were included in our meta-analysis on the basis of the following criteria: (1) the study was a cohort and/or case-control study evaluating the relationship between PD and risk of prostate cancer; (2) an estimate of association [e.g., incidence rate ratio, odds ratio, risk ratio (RR), hazard ratio or standardized incidence ratio] with measures of variation (i.e., confidence intervals, CI) was provided; (3) the study was published in English. When duplicated studies (based on the same population^32–36^) were identified, only the most informative study was included^10,20,21^. Case reports and abstracts from meetings were excluded.

Data from each study were extracted independently by two authors (Chunli Chen and Haiping Zheng) according to the Preferred Reporting Items for Systematic Reviews and Meta-Analyses (PRISMA) guidelines^37^, and any disagreements were resolved by discussion or involvement of a third reviewer if necessary. The following data were extracted from each included study: the first author, population country, study design (cohort or case-control), publication year, patient information (i.e., sample size, source, age and sex), ethnicity, follow-up time in years and outcome of interest. The Newcastle-Ottawa scale (NOS) was used to assess the quality of the included studies^38^. In addition, studies with NOS scores >6 were considered high-quality studies (Table S1).

### Statistical analysis

The pooled RRs and their 95% confidence intervals (CI) were calculated using the method of inverse variance with a random-effects model. Statistical heterogeneity was evaluated using Cochran’s Q test and I^2^ statistic^39^. Begg’s and Egger’s tests were used to evaluate the effect of publication bias^40,41^. Cumulative meta-analysis was used to assess the evolution of the combined RR in relation to the year of publication^42^. STATA software (version 13.0; Stata Corporation, College Station, TX, USA) was used to perform the data analyses in this study. A result was considered statistically significant when its P value was less than 0.05.

## Conclusions

In summary, the result of this study indicated that PD was significantly associated with a reduced risk of prostate cancer in the Western population. Future studies are warranted to confirm the association between those two conditions in the Asian population.

## Electronic supplementary material

Supplementary Information for Association between Parkinson's disease and risk of prostate cancer in different populations: An updated meta-analysis
