# Supplementary material for: Association between Parkinson’s disease and risk of prostate cancer in different populations: An updated meta-analysis
Source: Sci Rep. 2017 Oct 18;7:13449. doi: 10.1038/s41598-017-13834-x (PMC5647429; doi:10.1038/s41598-017-13834-x)
Supplement: Supplementary file 1 — Supplementary Information for Association between Parkinson's disease and risk of prostate cancer in different populations: An updated meta-analysis [file 41598_2017_13834_MOESM1_ESM.pdf]

Supplementary Information for Association between Parkinson's disease and risk of prostate cancer in different populations: An updated meta-analysis

Title of manuscript:
Association between Parkinson's disease and risk of prostate cancer in different populations: An updated meta-analysis

Chunli Chen, MD, Haiping Zheng, MD, Zhiping Hu, MD.
Department of Neurology, Second Xiangya Hospital, Hunan, P.R. China

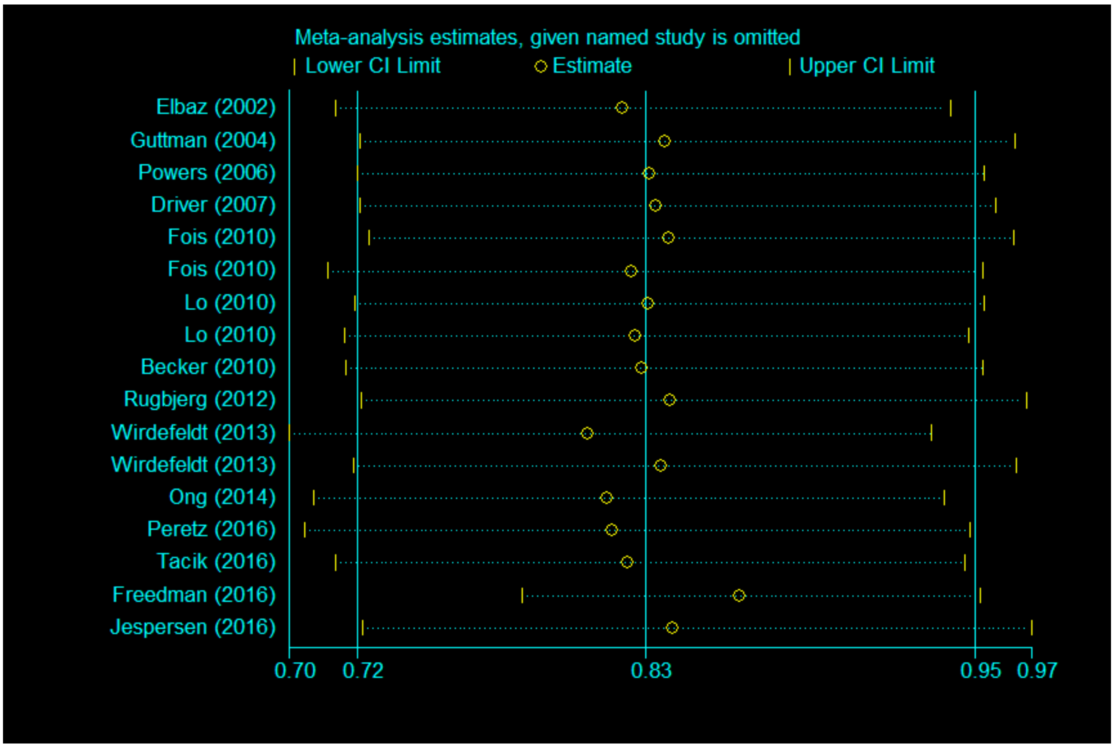

Figure S1: Sensitivity analysis for the association between Parkinson’s disease and risk of prostate cancer in Western population.

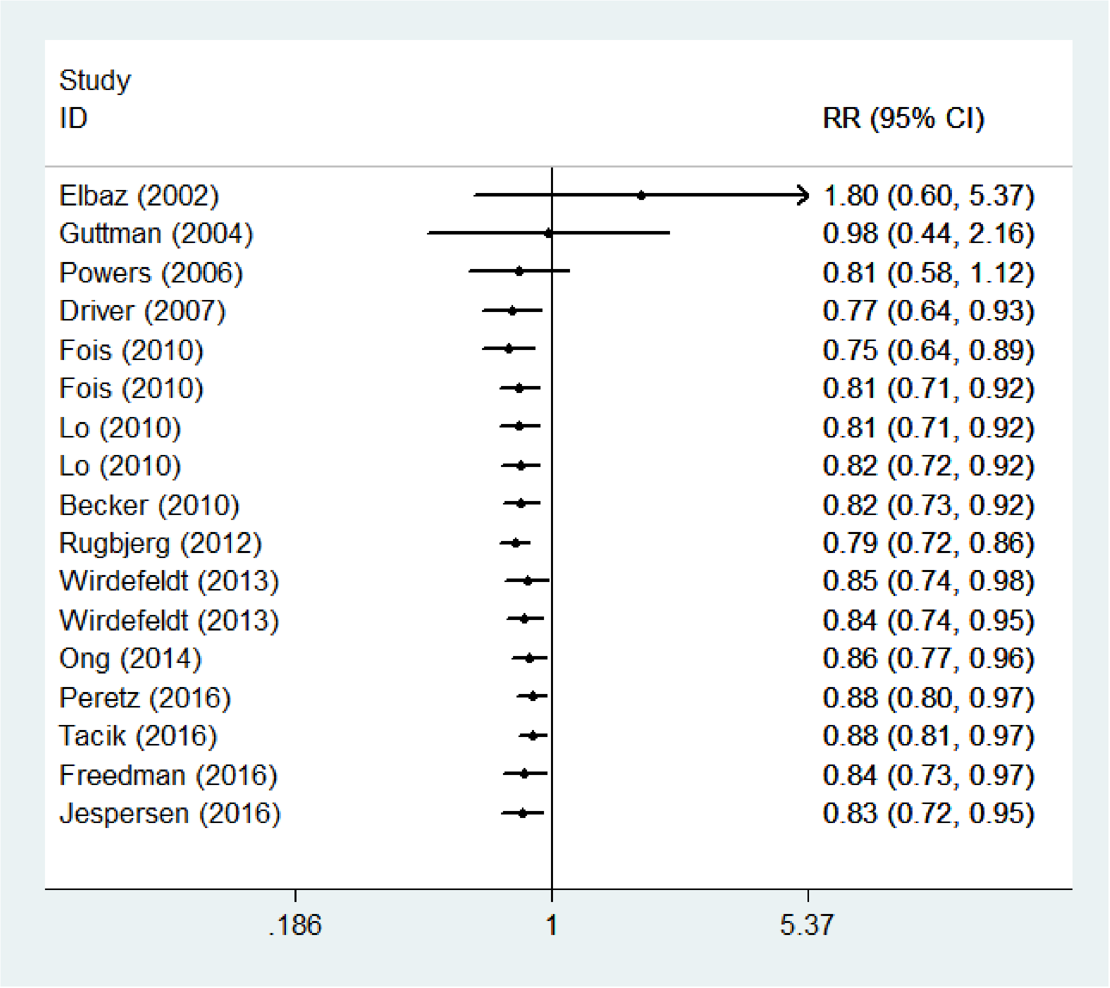

Figure S2: Cumulative meta-analysis or the association between Parkinson's disease and risk of prostate cancer in Western population.

A B

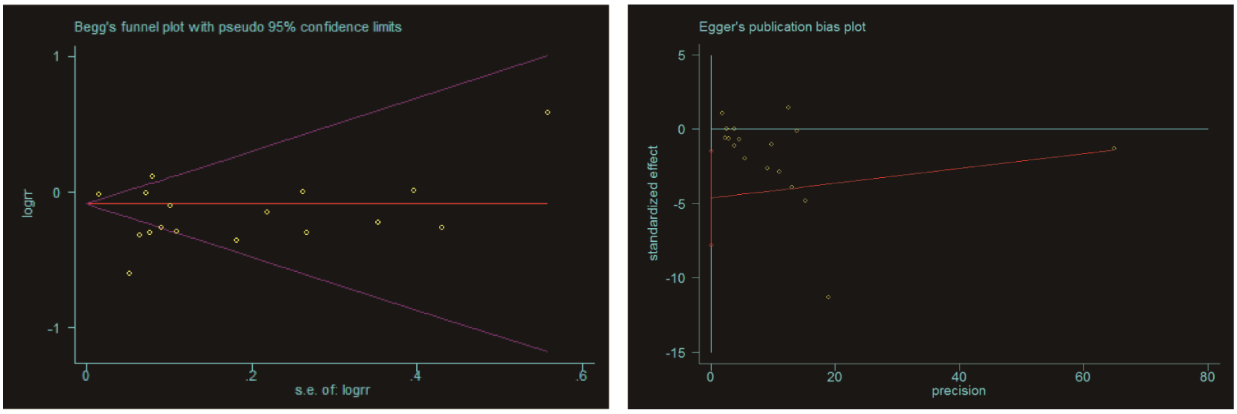

Figure S3: Test result for publication bias. (A) Begg's test; (B) Egger's test.

Study/ year	Selection				Control for important factors*	Exposure			NOS Score
	Adequate definition of cases	Representa tiveness of cases	Selection of controls	Definiti on of controls		Ascertainment of exposure	Same method to ascertain for cases and controls	Nonresponse rate	
Elbaz 2002	1	1	1	1	1	1	1	0	7
Guttman 2004	1	0	1	1	1	1	1	0	6
Powers 2006	1	1	0	1	1	1	1	0	6
Driver 2007	1	1	1	1	1	0	1	0	6
Fois 2010	1	0	1	1	2	1	1	0	7
Lo 2010	1	1	1	1	2	1	0	0	7
Becker 2010	1	1	1	1	1	0	1	0	6
Rugbjerg2 012	1	0	1	1	1	1	1	1	7
Wirdefeldt 2013	1	1	1	1	1	1	1	0	7
Ong 2014	1	1	1	1	1	1	1	1	8
Lin 2015	1	1	1	1	1	1	1	0	7
Peretz 2016	1	1	1	0	1	1	1	1	7
Tacik 2016	1	1	1	0	0	1	1	0	5
Freedman 2016	1	1	0	0	1	1	1	0	5
Jespersen 2016	1	1	1	1	1	1	1	0	7

Table S1. The quality of the included studies assessed by NOS.*: a maximum of two stars can be allotted in this category, one for age, sex, the other for other important controlled factors.
